# Hypomagnesaemia induced recurrent cerebellar ataxia: an interesting case with successful management

**DOI:** 10.1186/s40673-019-0110-9

**Published:** 2020-01-08

**Authors:** Singh Saraj Kumar, Goel Khushbu, Mukherji Joy Dev

**Affiliations:** 1 Department of Neurosurgery, AIIMS Patna, Patna, Bihar 801507 India; 2 Department of Neurology, Max Super Speciality Hospital, Saket, New Delhi, 110017 India

**Keywords:** Hypomagnesaemia, Cerebellum, Paraneoplastic syndrome, Proton pump inhibitors

## Abstract

**Purpose:**

Severe Hypomagnesaemia is a rare biochemical findings utilized for identifying the etiology of cerebellar ataxia. It requires a high degree of suspicion to diagnose. MRI findings are often nonspecific.

**Methods:**

The author presents a case of 38 yrs. old male patient presented with vomiting, gait imabalance and nystagmus. Biochemical investigations lead to severe hypomagnesaemia. Also MRI findings were matched suggesting of hyperintesity in left cerebellar hemisphere.

**Results:**

Patient was treated with magnesium infusion which leads to recovery of patient. Again the same symptomology was repeated after 3 months and disappearance after same treatment. Offending cause was diagnosed and proton pump inhibitors stopped.

**Conclusion:**

Severe Hypomagnesaemia is a rare but treatable cause if diagnosed at right time. It requires a high degree of suspicion to diagnose it. Measurement of serum magnesium levels should always be kept in back of mind if definite management of cerebellar symptoms has to be done.

## Introduction

Magnesium is a critical intracellular cation which is useful in many steps of enzymatic reactions. Its deficiency leads to many clinical features of neurotoxicity and cardiac failure as it regulate neurotransmission and stabilizes vascular endothelium [1, 2].Hypomagnesaemia induced neurological features include vertigo, chorea, tremors and fasciculations [3–5]. Critically low levels of magnesium can result in endovascular epithelium dysfunction that can present with clinical picture resembling posterior reversible encephalopathy. Cerebellar dysfunction may be primary manifestation of hypomagnesaemia. However its improvement on correction of serum magnesium level is rarely reported. The author is describing a young patient presented with cerebellar syndrome associated with hypomagnesaemia, which got corrected on normalizing the magnesium level.

## Case report

A 38 years old gentleman brought to emergency room with complaints of recurrent vomiting for 7 days, dizziness for 5 days, and gait imbalance along with visual perception of oscillatory movements of objects for last 2 days. There was no history of tremors/ diplopia/motor deficits/ or altered sensorium or seizures. History of fever/ chronic drug exposure/ thyroid disorder/Jaundice was absent. There was no history of alcohol consumption. Hypertension was present but controlled for last 1 year. There was history of receiving influenza vaccine 1 week back. On examination, Pt was alert with normal higher mental functions. Vitals were stable. Cranial nerve examination revealed bilateral downbeat nystagmus. Kayser Fleischer (KF) ring was absent. Fundus was normal. There was no motor or sensory deficits and reflexes were normal. Cerebellar examination revealed dysdiadokinesia, impaired finger nose finger test, impaired heel shin test. Gait was ataxic with left sided deviation.

Initial Lab investigations revealed normal complete blood count, (Liver function test) LFT, (Kidney Function Test) KFT with normal, (thyroid peroxidase) TPO Antibodies. Thyroid Stimulating hormone (TSH), Free triiodothyronine (FT3) and Free Throxine (FT4) were normal. Serum Mg was low, ***0.25 mg/dl*** (1.8–2.5). Serum calcium, Potassium and albumin were normal. Leptospira and chikangunya serology was negative. Serum Angotensin converting enzyme (ACE) was normal (13.1 U/L). Serum Vitamin B12 levels were in normal range. Viral markers were negative. Vasculitic and autoimmune profiles were negative. Paraneoplastic Profile result showed positive anti YO (qualitative) antibody. Fluorodeoxyglucose (FDG)-positron emission tomography (PET) scan was normal. Cerebrospinal fluid (CSF) analysis was done which was normal (Cells: 5 (all lymphocytic) Protein: 126 mg/dl, Glucose: 56 mg/dl (Blood sugar: 90 mg /dl)).

**Fig. 1:**
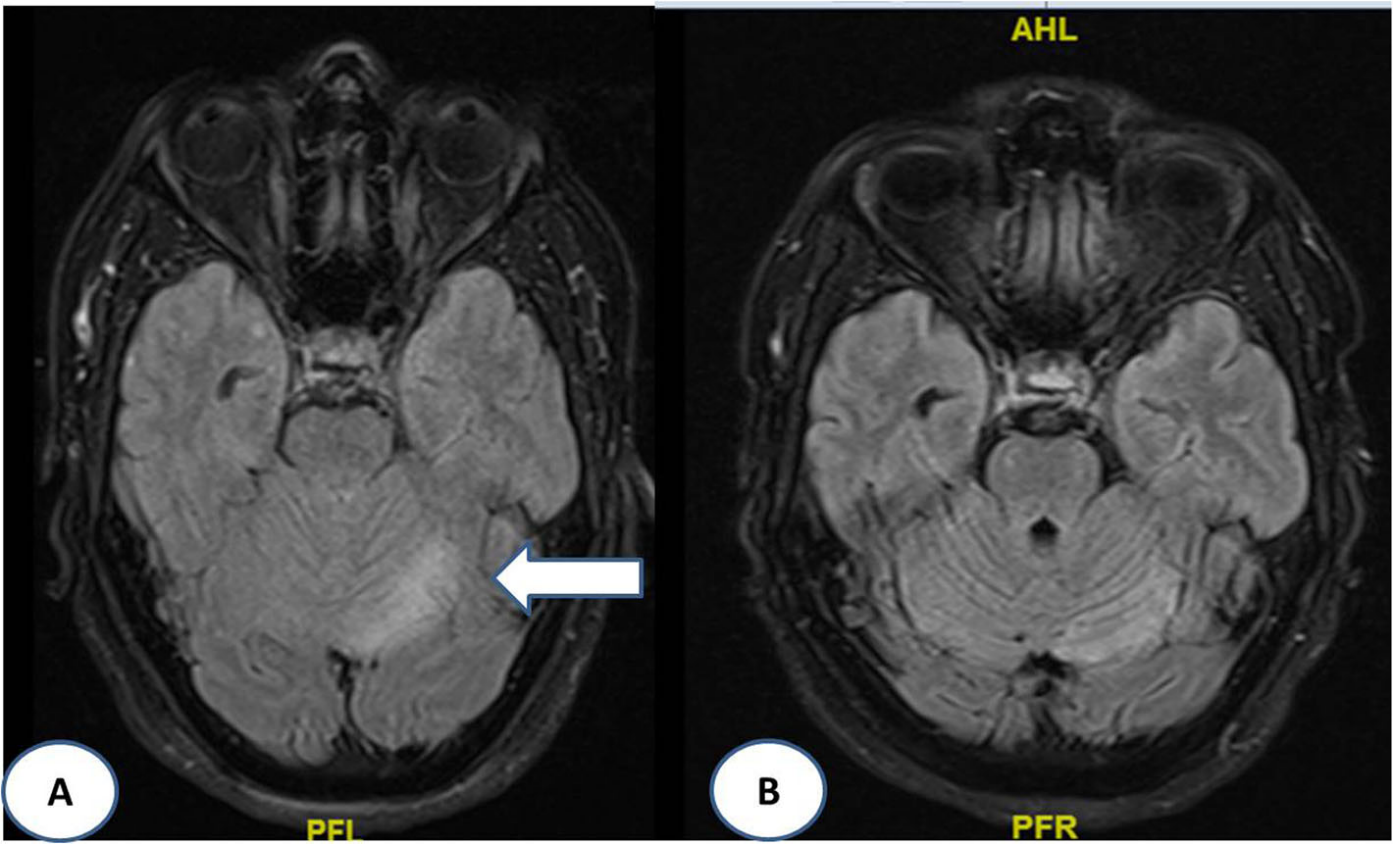
**a** MRI (axial), FLAIR sequence showing hyperintesity at left cerebellum. **b** MRI (axial), FLAIR sequence showing disappearance of hyperintesity

Treatment started with intravenous magnesium after which imbalance and vertigo improved. Pt was discharged on maintenance dose of magnesium. After 3 months, pt. came back with multiple episodes of whole body stiffness, uprolling of eyes, vigorous shaking, irritability, Short term memory loss, night time hallucinations. This time Serum magnesium levels were ***0.9 mg/dl***. Magnesium was replaced again this time, pt. improved gradually. Work for recurrent hypomagnesaemia was done which was negative. Patient was on ***proton pump inhibitors***. On stopping it, patient improved significantly. Magnetic Resonance Imaging (MRI) brain at the time of admission was showing hyperintensity at left cerebellar hemisphere which disappeared after 4 weeks of definite management. (Fig. 1a and b).

## Discussion

Magnesium is involved in neuromuscular excitability and cell permeability, as well as the regulation of ion channels and mitochondrial function. Various hypomagnesaemia-associated symptoms involving neuromuscular hyper excitability are observed when serum levels fall below 1.0 mg/dL [2].The present patient with cerebellar syndrome had recurrent episodes of severe hypomagnesaemia along with positive ***anti yo antibody***. Initially the diagnosis of ***Para neoplastic cerebellar degeneration with unknown primary*** was made in view of **young age and positive serum** anti yo antibody. But low serum magnesium level along with immediate recovery after intravenous magnesium diminishes the diagnosis of Para neoplastic encephalitis. The neuroimaging findings and its reversal in our patient are more consistent with the clinical syndrome of reversible posterior leukoencephalopathy syndrome (PRES). But this condition is generally seen with ***hypertensive emergencies*** [3, 5].In our patient blood pressure was normal throughout the management and there was no history of any antihypertensive drugs. There are case reports suggesting similarities between PRES and severe hypomagnesaemia [3, 5].In these syndromes, it is believed that the auto regulation capacity of the posterior circulation vascular endothelium is overridden, resulting in oedematous changes and cerebral dysfunction especially vertigo, nystagmus, aphasia, hemiparesis, depression, delirium, choreoathetosis [3, 5].So it is very essential to look for reversible causes of cerebellar syndrome, especially hypomagnesaemia so that patients can be treated effectively. Wernicke’s encephalopathy also causes cerebellar signs. But in the presence of severe hypomagnesaemia, intravenous thiamines will not respond [6].

However; studies in last decade have suggested that continuous utilization of ***Proton pump inhibitors*** can lead to severe degree of hypomagnesaemia causing cerebellar symptoms. Our patient was taking proton pump inhibitors for last many months which lead to this amount of hypomagnesaemia. Low levels of magnesium also cause falling of serum calcium and phosphate, which ultimately disturbs body cellular activity and neuromuscular excitability [7, 8].

This rare case report reveals importance of out of way thinking by clinicians at an appropriate time regarding importance of magnesium in various body regulations. Magnesium is much underrated cation. Its serum levels are very rarely performed for ruling it out as one of the etiologies for neurological manifestations especially cerebellar symptoms.

## Conclusion

Although hypomagnesaemia is one of the rare causes for cerebellar symptoms, but during acute phase, monitoring of magnesium levels should always be kept in mind. Correction of reversible causes like hypomagnesaemia always improves both clinical and radiological features. Careful history of ongoing and previous medications especially ***proton pump inhibitors*** should always be taken during recurrent exacerbations of cerebellar symptoms.

## Data Availability

Available.

## Abbreviations

CSF: Cerebrospinal fluid
FDG PET scan: Fluorodeoxyglucose (FDG)-positron emission tomography (PET)
FT3: Free triiodothyronine
FT4: Free Thyroxine
KFT: Kidney Function Test
LFT: Liver function Test
MRI: Magnetic Resonance Imaging
PRES: Posterior Reversible Encephalopathy Syndrome
TPO: Thyroid peroxidase
TSH: Thyroid Stimulating hormone
